# Age-Related Changes in the Epithelial and Stromal Compartments of the Mammary Gland in Normocalcemic Mice Lacking the Vitamin D_3_ Receptor

**DOI:** 10.1371/journal.pone.0016479

**Published:** 2011-01-26

**Authors:** JoEllen Welsh, Lindsay N. Zinser, Laurel Mianecki-Morton, Jamie Martin, Susan E. Waltz, Howard James, Glendon M. Zinser

**Affiliations:** 1 University at Albany Cancer Research Center, Rensselaer, New York, United States of America; 2 Cincinnati VA Medical Center, Cincinnati, Ohio, United States of America; 3 University of Michigan, Ann Arbor, Michigan, United States of America; 4 Brown University/Hasbro Children's Hospital, Providence, Rhode Island, United States of America; 5 University of Cincinnati, Cincinnati, Ohio, United States of America; 6 Cincinnati Shriner's Hospital, Cincinnati, Ohio, United States of America; The University of Hong Kong, Hong Kong

## Abstract

The vitamin D_3_ receptor (VDR) serves as a negative growth regulator during mammary gland development via suppression of branching morphogenesis during puberty and modulation of differentiation and apoptosis during pregnancy, lactation and involution. To assess the role of the VDR in the aging mammary gland, we utilized 12, 14, and 16 month old VDR knockout (KO) and wild type (WT) mice for assessment of integrity of the epithelial and stromal compartments, steroid hormone levels and signaling pathways. Our data indicate that VDR ablation is associated with ductal ectasia of the primary mammary ducts, loss of secondary and tertiary ductal branches and atrophy of the mammary fat pad. In association with loss of the white adipose tissue compartment, smooth muscle actin staining is increased in glands from VDR KO mice, suggesting a change in the stromal microenviroment. Activation of caspase-3 and increased Bax expression in mammary tissue of VDR KO mice suggests that enhanced apoptosis may contribute to loss of ductal branching. These morphological changes in the glands of VDR KO mice are associated with ovarian failure and reduced serum 17β-estradiol. VDR KO mice also exhibit progressive loss of adipose tissue stores, hypoleptinemia and increased metabolic rate with age. These developmental studies indicate that, under normocalcemic conditions, loss of VDR signaling is associated with age-related estrogen deficiency, disruption of epithelial ductal branching, abnormal energy expenditure and atrophy of the mammary adipose compartment.

## Introduction

The developmental changes associated with puberty, pregnancy, lactation and involution of the mammary gland have been extensively studied and signaling pathways initiated by both membrane and nuclear receptors are essential for coordination of these events [1]. However, less is known about the pathways that control turnover of the mammary cell populations during the aging process. In mice, ovariectomy (to mimic the post-menopausal state) is associated with cessation of mammary epithelial cell proliferation, which can be restored by treatment with estrogen and progesterone, although the sensitivity of the gland to these hormones decreases with age [2], [3]. While estrogen is clearly needed for maintenance of the ductal epithelium, it does not appear to be required for development or maintenance of the adipose/stromal compartment [4].

Stromal-epithelial interactions are crucial for mammary gland development and maintenance, including the mammary fat pad, which provides signals that mediate ductal morphogenesis [5], [6]. The mammary adipose tissue functions as an endocrine organ, producing steroid and peptide hormones as well as hormone-like molecules known as adipokines [7], [8], which impact on the mammary epithelial compartment. The total absence of white adipose tissue in the mammary gland disrupts stromal-epithelial interactions and prevents normal mammary gland development [6], [9]. Thus, when investigating the development or homeostasis of the mammary epithelium, it is necessary to consider all microenvironments of the mammary gland.

We have previously shown that the nuclear vitamin D_3_ receptor (VDR), whose ligand, 1,25-dihydroxyvitamin D_3_ (1,25D), a derivative of vitamin D_3_, is expressed and dynamically regulated in mammary gland during the reproductive cycle [10]. VDR agonists have been shown to modulate proliferation and survival of stromal and epithelial cells derived from mammary gland, and can inhibit growth of breast cancers in animal models [11], [12], [13], [14]. Furthermore, VDR knockout (KO) mice exhibit accelerated mammary gland development during puberty and early pregnancy, and impaired apoptosis during involution, compared to wild-type (WT) mice [10], [15]. Although the VDR is essential for intestinal calcium absorption, the effects of VDR ablation on mammary gland were observed in mice maintained on a high calcium rescue diet which normalizes serum calcium, bone growth, and fertility [15], [16], [17], indicating that the effects of VDR on mammary gland represent calcium-independent actions. Additional novel functions of VDR that have been uncovered using the normocalcemic VDR KO mouse model include effects on the immune system [18], the renin-angiotensin system [19], adipogenesis [20], [21] and tumorigenesis [22].

In the studies reported here, we used normocalcemic VDR KO mice to determine whether the changes we observed in the mammary gland during pubertal development and the reproductive cycle of VDR KO mice persist or are exacerbated with age. We hypothesized that chronic absence of vitamin D_3_ signaling, via VDR ablation, in the mammary gland might impact ductal epithelial cell turnover, leading to hyperplastic nodules that could lead to transformation. We demonstrate that VDR expression persists in the aging mammary gland of WT mice, but in contrast to expectations, abnormal energy metabolism in older VDR KO mice leads to atrophy of the mammary adipose compartment and apoptotic regression of the mammary epithelium. Thus, our studies suggest that VDR signaling is required for overall metabolic homeostasis and for maintenance of epithelial and stromal interactions in the mammary gland during the aging process.

## Results

### VDR expression persists in aging mammary gland

**Figure 1:**
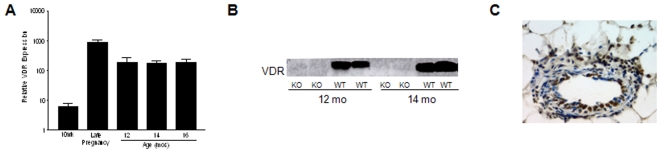
Expression of the VDR in aging mammary glands of WT mice. A. Real Time PCR for VDR gene expression in aging mammary glands (12, 14, and 16 months) derived from wild type (WT) mice were compared to mature 10 week (10wk) pubertal and late pregnancy mammary glands. Data are expressed relative to 18S RNA (Relative VDR Expression) and represent mean ± s.e.m. of triplicate runs. B. Western blot of VDR protein expression in aging mammary glands (12 and 14 months) from WT mice, which is undetectable in VDR KO glands. C. Formalin fixed sections of mammary gland from 12, 14, and 16 month old WT mice were subjected to immunohistochemistry with a monoclonal antibody directed against VDR. A representative stained section of a 12 month WT gland shows positive staining in the mammary epithelium, stroma and adipose, a pattern that is similar to pubertal mammary gland expression [15]. VDR positive cells appear brown against the blue hematoxylin counterstain.

Vitamin D_3_ deficiency is prevalent in the elderly population and has been linked to an increased risk for breast cancer [23]. To assess the potential impact of vitamin D_3_ signaling in aging mammary glands, we used real time PCR to assess VDR gene expression in mammary glands harvested from 12, 14 and 16 month old virgin mice. As shown in Figure 1A, VDR expression was detected in mammary glands from aging WT mice at levels slightly lower than that found during pregnancy, when expression of this receptor is highly induced [10], [24]. Interestingly, VDR mRNA in aging mammary gland was about 10-fold higher than that in post-pubertal, non-pregnant mice, where VDR promotes differentiation [15]. VDR protein was detected in aging mammary glands of WT mice but not in VDR KO mice by western blotting of tissue homogenates (Figure 1B). Immunohistochemistry was used to determine VDR localization within specific cell types of the gland of aging WT mice. Representative staining of the inguinal mammary gland from a 12 month old virgin WT mouse (Figure 1C) demonstrates positive staining for the VDR in the nuclei of ductal epithelial cells and in the stromal and adipose cells surrounding the ducts, similar to what has been reported previously in pubertal mammary glands from virgin WT mice [15].

### Effects of VDR ablation on mammary gland morphology

**Figure 2:**
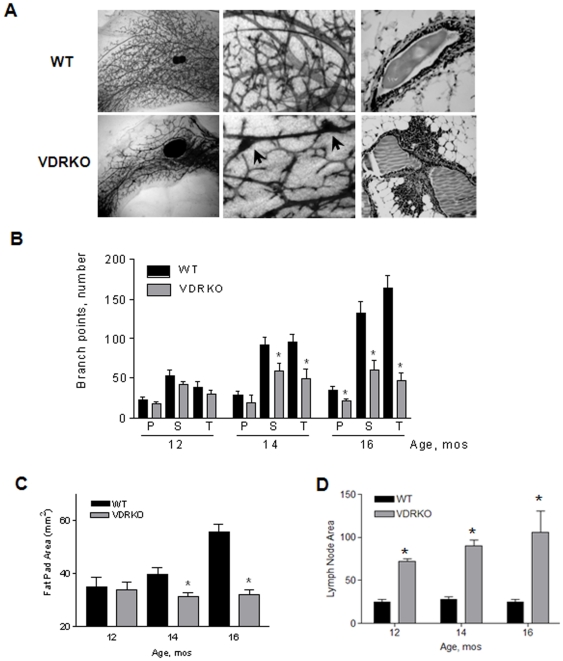
Morphological and quantitative assessment of inguinal mammary glands from 12, 14, and 16 month old WT and VDR KO mice. A. (Left Panels) Glands from VDR KO mice show decreased secondary and tertiary branching, signs of ductal thickening (ectasia), and large lymph nodes as the adipose tissue begins to atrophy with age. In contrast, WT glands show an extensive array of secondary and tertiary branches resting in a vast fat pad of plentiful adipose tissue. (Middle Panels) Glands at higher magnification from VDR KO mice show reduced secondary and tertiary branching and areas of darkly stained lesions along primary ducts (arrows). (Right Panels) Histological sections stained with hematoxylin and eosin Y show clusters of inflammatory cells along the primary ducts within glands of VDR KO mice compared to WT glands. B. Primary, secondary and tertiary branch points were quantitated as described in materials and methods. Primary branch counts showed a significant decrease between WT and KO glands at 16 months whereas secondary and tertiary branches were significantly decreased in glands from VDR KO mice at 14 and 16 months compared to WT control mice. *Statistically significant by Students t test, WT vs. VDR KO, n = 10–12 p<0.05. C. Whole mounts from VDR WT and KO mice were utilized to measure the area of mammary fat pads using AxioVision software. Fat pad area was calculated by tracing around the exterior of the ductal branches of the mammary fat pads. At 14 and 16 months of age, there is a significant reduction in the mammary fat pads of VDR KO mice compared to WT controls. *Statistically significant by Students t test, WT vs. VDR KO, n = 10–12 p<0.05. D. Whole mounts from VDR WT and KO mice were utilized to measure the mammary lymph nodes. Using AxioVision software, the exterior of the glandular lymph node was traced to calculate the area. At all time points, lymph nodes within glands from VDR KO mice were significantly enlarged compared to lymph nodes from WT mice. *Statistically significant by Students t test, WT vs. VDR KO, n = 10–12 p<0.05.

To assess the long-term impact of VDR ablation on mammary gland ductal morphology, whole mounts of inguinal mammary glands were prepared from virgin WT and VDR KO mice sacrificed between 6 and 16 months of age. Although the gross morphology of mammary glands from WT and VDR KO mice at 6, 8 and 10 months were similar (data not shown), developmental differences were apparent in glands from VDR KO mice beginning at 12 months. Representative micrographs of inguinal mammary glands from 16 month old mice are shown in Figure 2A. Whole mounted glands (left panels) from WT mice displayed dense ductal extension past a small central lymph node and extensive secondary and tertiary branching morphogenesis. Although the glands from VDR KO mice showed normal ductal extension, the extent of epithelial branching was significantly reduced and the lymph nodes were markedly enlarged. At higher magnification (middle panels), the degree of secondary and tertiary branching was consistently reduced in glands from VDR KO mice. In addition, darkly stained lesions were frequently observed along the primary ducts of glands from VDR KO mice (Figure 2A, lower middle panel, arrows). On H&E preparations (Figure 2A, right panels), these lesions, which were present only in VDR KO mice, corresponded to dense clusters of inflammatory cells adjacent to the ducts, particularly at branch points.

Counts of primary, secondary and tertiary branch points (Figure 2B) indicated that the increase in secondary and tertiary ductal branching evident with age in WT mice was retarded in VDR KO mice. In addition to the differences in epithelial structure, the increase in mammary fat pad area that occurred with age in WT mice did not occur in VDR KO mice (Figure 2C). In fact, the mammary adipose tissue size decreased with age in VDR KO mice, and by 16 months, the average fat pad area in VDR KO mice was less than half the size of their WT counterparts. In contrast, the size of the central lymph node was significantly increased in VDR KO animals over the aging time course (Figure 2D).

**Figure 3:**
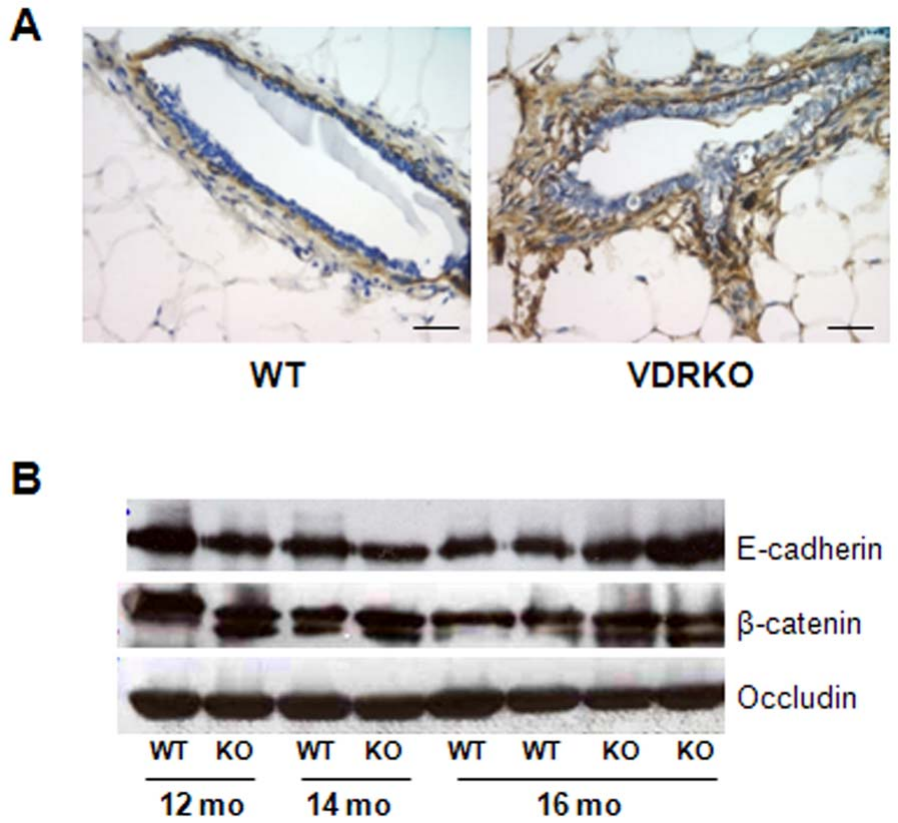
Stromal smooth muscle actin and cell junction expression in glands from WT and VDR KO mice. A. Elevated level of smooth muscle actin staining in VDR KO glands compared to VDR WT control sections. SMA positive cells appear brown against the blue hematoxylin counterstain. B. Western blot for cell junction markers E-cadherin, β-catenin, and occludin to assess changes in the epithelial cell junctions that may account for the increase in secretion within the VDR KO ducts. E-cadherin, β-catenin, and occludin expression were equivalent at all time points during aging development between VDR WT and KO mammary glands.

At the histological level, sections were stained for smooth muscle actin (SMA) to determine whether VDR ablation altered the stromal compartment of the mammary gland. As shown in Figure 3A, increased SMA staining surrounding the ducts of VDR KO mice was consistently observed compared to WT controls. Since vitamin D_3_ has been shown to regulate E-cadherin in mammary cells in vitro, we assessed the expression of several proteins associated with the epithelial tight junctions. No differences in E-cadherin, β-catenin or occludin abundance were detected by western blotting of tissue extracts from WT and VDR KO mice at any age (Figure 3B).

### Effect of VDR ablation on adiposity and energy expenditure

**Figure 4:**
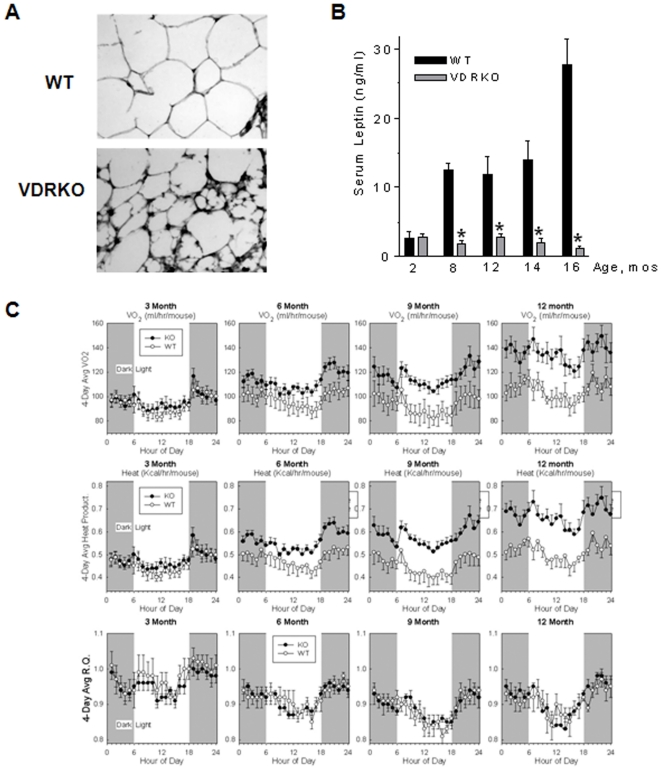
Adipose morphology and indirect calorimetric assessment throughout the aging of VDR WT and KO mice. A. Histological sections stained with hematoxylin and eosin Y show the small multi-locular adipocytes in glands of 16 month VDR KO mice compared to the large uni-locular fat cells in WT mice. B. Serum leptin was assessed by ELISA. There was a significant reduction in serum leptin by 8 months of age in the VDR KO mice that persisted through 16 months of age as a result of WT mice experiencing a progressive elevation in serum leptin beginning at 6 months and peaking at 16 months. C. Indirect calorimetric measurements were conducted every three months (3–12 months) in VDR WT and KO mice. Average oxygen consumption (VO_2_) was trending higher by 6 months in VDR KO mice and was significantly elevated at 9 and 12 months compared to VDR WT mice. n = 6 p<0.05. Energy expenditure (Heat Kcal/hr/mouse) was similar to VO_2_ in that VDR KO mice experience a significant elevation in energy expenditure by 9 and 12 months. n = 6 p<0.05. Respiratory exchange ratio (RQ), which infers the energy source utilized by the animal, was equivalent between VDR WT and KO mice throughout the aging time course (3–12 months).

To further explore the effect of VDR signaling on the stromal compartment, the cellularity of the mammary fat pad was compared in WT and VDR KO mice. As shown in Figure 4A, the mammary fat pads from 16 month WT mice were primarily composed of large uni-locular fat cells, whereas the adipose compartment of VDR KO mice contained smaller adipocytes, many of which were multi-locular and histologically reminiscent of brown adipocytes. To confirm previous reports of reduced whole body adiposity in younger VDRKO mice [20], we measured serum concentrations of leptin, a cytokine secreted from mature adipocytes which reflects overall body fat stores. Serum leptin (Figure 4B) was significantly lower in aging VDR KO mice compared to WT mice, showing a reduction as early as four months of age and significantly reduced by eight months, suggesting that the atrophy of the mammary fat pad in VDR KO mice reflects comparable decreases in total body fat stores.

To determine whether long-term VDR ablation altered global energy metabolism, we utilized indirect calorimetry to assess metabolic rate in male WT and VDR KO mice as a function of age. Male mice were used to help define the impact of VDR ablation on energy consumption without the underlying concern of hormonal fluctuations found in female mice during each estrous cycle and throughout the aging time course. Compared to WT mice, VDR KO mice exhibited an elevated VO_2_ level, indicating a hypermetabolic phenotype, beginning at 6 months of age, with significant (p<0.05) differences detected from 9 months of age on (Figure 4C, top panel). Energy expenditure is also reflected as heat production, expressed as calories, which was elevated in the VDR KO mice beginning at 6 months of age and significantly higher by 12 months (Figure 4C, middle panel). Finally, indirect calorimetry also allows inference of the energy source (carbohydrate versus fat) based on the respiratory exchange ratio (RQ). The RQ data indicates no difference in energy source between WT and VDR KO mice over the 12 month time course (Figure 4C, bottom panel). Collectively, these data indicate that atrophy of the mammary fat pad in aging female VDR KO mice reflects a reduction in whole body fat stores secondary to enhanced energy expenditure.

### VDR KO mice are normocalcemic but display reduced circulating estrogen

**Figure 5:**
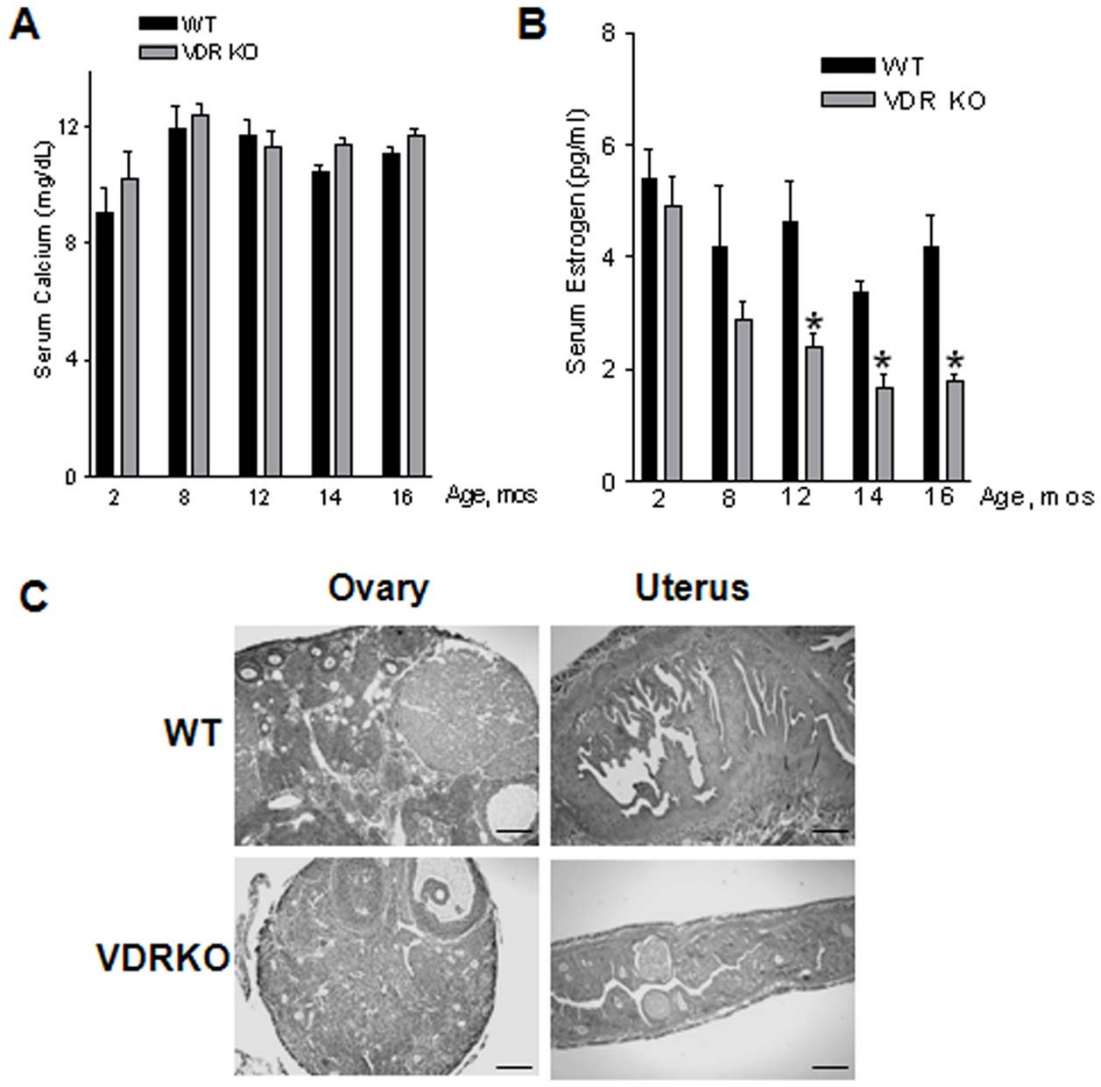
Effect of VDR ablation on serum calcium, estrogen, and estrogen responsive tissues in aging WT and VDR KO mice. A. Serum calcium levels in aging WT and VDR KO mice were equivalent prior to (2 and 8 months) and throughout the aging time course (12–16 months). B. Serum estrogen was measured by radioimmunoassay in WT and VDR KO mice at 2, 8, 12, 14, and 16 months of age. There was a significant reduction in serum estrogen by 12 months of age in the VDR KO mice that persisted through 16 months of age. *Statistically significant by Students t test, WT vs. VDR KO, n = 8–10 p<0.05. C. Representative hematoxylin and eosin Y stained sections of the ovary and the uterus, an estrogen responsive tissue. VDR KO tissues show signs of atrophy, likely due to incomplete follicle formation in the ovary and thus decreasing serum estrogen and inducing atrophy in the uterus compared to WT ovary supporting follicle formation and a responsive and robust uterus. Scale bar: 200 µm.

Vitamin D is a critical regulator of calcium homeostasis, and VDR KO mice require a high calcium “rescue” diet during early development to prevent skeletal defects and maintain fertility [16], [25]. However, it is unclear whether this rescue diet can maintain normocalcemia over the lifespan of VDR KO mice. Since calcium has been linked to body weight regulation [26], [27], it was important to determine whether the altered mammary gland morphology in VDR KO mice was associated with disturbances in calcium homeostasis. We therefore measured serum calcium as a function of age in WT and VDR KO mice compared to pubertal mice (2 months). As shown in Figure 5A, serum calcium was comparable at 2, 8, 12, 14, and 16 months of age in VDR WT and KO mice, suggesting that the high calcium rescue diet is sufficient to prevent hypocalcemia in VDR KO mice regardless of age.

We assessed whether the effect of VDR ablation on glandular maintenance was related to alterations in estrogen signaling. The expression of estrogen or progesterone receptors in the mammary gland (as measured by immunohistochemistry and western blot) was not affected by VDR ablation (data not shown). However, serum 17β-estradiol was significantly reduced in 12, 14, and 16 month old VDR KO mice compared to age-matched WT mice and compared to pubertal aged WT and VDR KO mice (Figure 5B). Serum 17β-estradiol progressively decreased in the VDR KO mice beginning at 8 months of age whereas WT mice maintained a relatively constant 17β-estradiol serum level through 16 months of age. Histological analysis indicated that the decrease in serum 17β-estradiol in aged VDR KO mice resulted from ovarian failure, as there was little evidence of follicle formation in the ovaries of VDR KO mice from 12 months of age on (Figure 5C, left panels). By 16 months of age, the ovaries of VDR KO mice were about half the size of their WT counterparts. Furthermore, the uterus, another estrogen responsive tissue, displayed severe atrophy in aging VDR KO mice compared to WT controls (Figure 5C, right panels). Thus, decreased estrogen signaling due to ovarian atrophy likely contributes to the reduction in ductal branching characteristic of aging VDR KO mammary glands.

### Markers of apoptosis are increased in mammary glands from aging VDR KO mice

**Figure 6:**
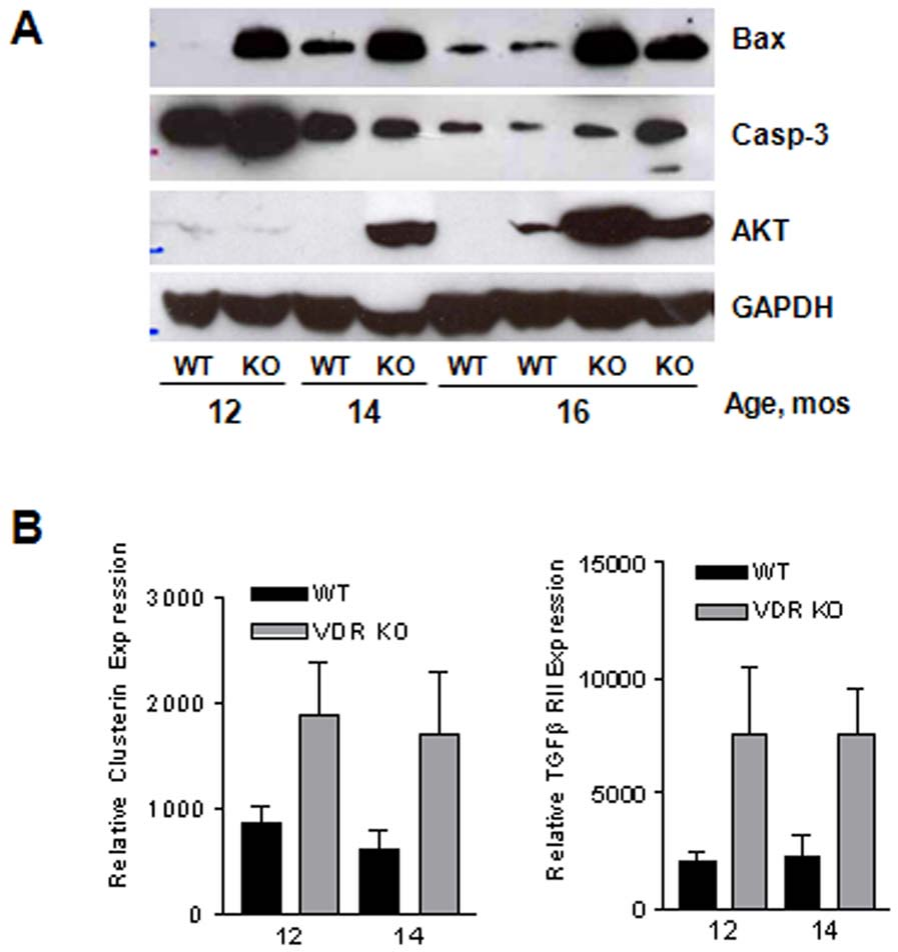
Assessment of apoptotic and survival related proteins in aging mammary glands from WT and VDR KO mice. A. Western blot of Bax, caspase-3, and AKT expression during aging development. VDR KO glands have a higher Bax expression at all aging time points, suggesting a possible increase in apoptosis in aging VDR KO glands. Caspase-3 and AKT expression are also elevated in VDR KO mice, particularly at 14 and 16 months. GAPDH serves as a loading control. B. Real Time PCR for Clusterin and TGFβII gene expression in aging mammary glands (12 and 14 months) derived from WT and VDR KO mice. Data are expressed relative to 18S RNA (Relative Gene Expression) and represent mean ± s.e.m. of triplicate runs.

The severe adipose tissue atrophy, altered stromal architecture and reduced branching in glands from VDR KO mice suggested an imbalance in mammary cell turnover. Therefore, we measured the expression of Bax and caspase-3, genes associated with apoptosis, in lysates of mammary tissue from WT and VDR KO mice. As shown in Figure 6A, Bax, a pro-apoptotic bcl-2 family member that is linked to physiologic apoptosis in the mammary gland [28], was minimally expressed in tissue from WT mice, but was consistently elevated in tissue of VDR KO mice. Caspase-3 expression and cleavage was detected in several samples from VDR KO mice, but this was not as consistent as bax elevation. Surprisingly, AKT, a gene that mediates cell survival, was also up-regulated in aging VDR KO tissue relative to WT tissue. Collectively these data may indicate that different cell populations in the gland respond differently to VDR ablation, and/or that cell survival signals are triggered in response to the tissue regression that occurs in the VDR KO mice.

Real time PCR was used to compare the expression of several genes linked to mammary gland differentiation and turnover, including beta casein, clusterin, fatty acid synthase, macrophage inhibitory factor, and TGFβRII. Expression of two genes, clusterin and TGFβRII, were found to be significantly up-regulated in VDR KO mice (Figure 6B). Induction of both clusterin and TGFβRII has been linked with apoptosis during post-lactational involution in the murine mammary gland, consistent with the concept that atrophy of aging VDR KO glands may involve apoptosis.

## Discussion

These studies have revealed a novel, age-related contribution of the VDR in maintenance of mammary gland integrity and adiposity. Through comparative analysis of mammary gland development and gene expression in relation to age, we report that mice lacking the VDR exhibit ductal ectasia, reduced branching and progressive atrophy of the mammary fat pad with age. These changes do not become evident until the onset of the second year of life and are in sharp contrast to the effects of VDR ablation observed during puberty and pregnancy, when lack of the VDR is associated with accelerated branching and precocious alveolar development [10], [15]. Importantly, the altered mammary gland development observed in both young and aged VDR KO mice occurred in the absence of hypocalcemia, indicating that these effects of VDR ablation are not secondary to the role of VDR in maintenance of extracellular calcium homeostasis.

Due to the chronic and systemic nature of VDR ablation in these studies, the extent to which VDR signaling within the mammary gland is responsible for maintaining glandular homeostasis is unclear. In support of direct effects of VDR signaling, VDR expression remained high in the aging mammary gland, and positive VDR staining was observed in glandular epithelial and stromal cells as well as adipocytes. Although the effects of 1,25D and VDR on mammary epithelial cells have been well studied, little attention has been paid to the effects of VDR on fibroblasts or adipocytes. Thus, the dramatic changes in the stromal microenvironment of the mammary gland of VDR KO mice were unexpected. The absence of VDR signaling in the adipocytes and stromal fibroblasts may contribute to the regression of ductal branches, since stromal-derived growth factors and extracellular matrix proteins are crucial for normal mammary gland development and maintenance [29], [30]. In addition, mice lacking white adipose tissue have short and severely distended ducts (ductal ectasia) [6], [9] similar to those observed in aged VDR KO mice. Deciphering the specific role of VDR signaling within each cell population will be necessary to elucidate the contributions and mechanisms of VDR signaling in maintenance of glandular homeostasis.

Regardless of mechanism, our data indicate an elevation in apoptotic signaling, including activation of the standard apoptotic markers Bax and Caspase-3 and up-regulation of clusterin and TGFβRII, in VDR KO mammary tissue. Since glandular homogenates were used for these assays, further studies will be needed to determine whether apoptosis is increased in the epithelium, the stroma or both compartments of aging VDRKO mice. We also observed an increase in AKT expression, which may be an adaptive response to the elevated apoptosis, since AKT activation of phosphodiesterase 3B limits cAMP production and reduces protein kinase A (PKA) activity. These changes inhibit lipolysis since PKA activity is required for phosphorylation and activation of hormone sensitive lipase [31]. Therefore, AKT may be elevated in mammary adipose tissue of VDR KO mice in an effort to block further hydrolysis of triglycerides. Characterization of the metabolic disturbances resulting from VDR ablation specifically within the adipose tissue will be necessary to clarify the significance of these findings.

Our work reports that the depletion of adipose stores observed in younger VDR KO mice [20], [21] progresses with age and results in severe atrophy of the mammary fat pad in parallel with age-related elevations in respiration rate and energy expenditure. A previous study by Wong et al. [21], reported minimal differences in energy expenditure between WT and VDRKO male mice unless they were challenged with a high fat diet, however, the age at which energy expenditure measurements were done in that study was not stated. In our mice on the low fat rescue diet, changes in energy metabolism secondary to VDR ablation were evident within 6 months, progressed with age and correlated with up-regulation of uncoupling protein-1 in adipose tissue [20] and mammary gland (Narvaez and Welsh, unpublished). Collectively, these data indicate that VDR signaling is necessary for suppression of uncoupling protein-1 and control of energy metabolism in both visceral and subcutaneous adipose depots, including maintenance of the stromal microenvironment of the mammary gland and ultimately ductal branching and epithelial cell survival.

Our studies also provide additional insight into the interactions between VDR, ovarian function and fertility. Neither fertility nor circulating estrogen is compromised in young VDRKO mice maintained on the high calcium rescue diet [10], [15], [16], however, here we demonstrate that VDR is necessary for maintenance of ovarian function and estrogen production with age. Since VDR is expressed in granulosa and corpus luteal cells [32], the ovarian failure we observed in mice 12 months and older may represent loss of VDR regulated gene expression within ovarian tissue. Regardless of mechanism, our data indicates that premature menopause represents another manifestation of accelerated aging in VDRKO mice. Clearly, an age-related decline in estrogen availability could contribute to the reduced branching in the mammary gland of VDR KO mice, since involution of side branches and loss of branch points occurs within five weeks of ovariectomy in mouse models of menopause [33]. However, ovarian failure is unlikely to contribute to the mammary fat pad atrophy in the VDRKO mice since chronic estrogen deficiency secondary to ablation of either estrogen receptor alpha or aromatase leads to accumulation of adipose tissue [34], [35]. Furthermore, we observed disturbed adiposity and altered energy metabolism in male VDR KO mice, suggesting these effects are independent of estrogen deficiency. Thus, while ovarian failure likely contributes to the activation of apoptosis and subsequent reduction in epithelial branching in the mammary gland, it does not explain the adipose tissue atrophy in VDR KO mice. Further studies will clearly be necessary to fully define the interactions between VDR signaling, metabolism, adipose and aging.

Our studies also identify a novel anti-inflammatory effect of VDR signaling in the mammary gland. Inspection of mammary gland whole mounts at high magnification revealed multiple dense lesions in VDR KO mice that were not present in WT mice. Although these lesions were initially thought to be hyperplastic epithelial nodules, Hematoxylin and Eosin Y staining indicated the presence of inflammatory cells, in support of chronic inflammation in VDR KO mammary tissue. Furthermore, the inguinal lymph nodes of VDR KO mice were significantly larger than those of WT mice. Our findings are consistent with reports that VDR KO mice exhibit altered cytokine profiles, T cell populations and antibody responses, and are highly susceptible to colonic inflammation with age [36], [37].

In summary, chronic VDR ablation exerts global effects on energy metabolism and ovarian function that are associated with alterations in mammary gland, including atrophy of the fat pad, degeneration of epithelial ductal branching, enlarged lymph nodes and chronic inflammation. These progressive changes represent additional features of the accelerated aging phenotype of VDR KO mice, and are distinct from the effects of VDR ablation during puberty and pregnancy. Other phenotypic changes that become evident with age in VDRKO mice include alopecia [17], wrinkling of the skin [24], [38], hearing loss [39], osteoblast differentiation failure [40], altered immunity [36] and hematopoietic disturbances [41]. The mechanisms underlying accelerated aging in VDRKO mice are unclear, and with the exception of alopecia (which represents a 1,25D independent effect of VDR), the role of the VDR ligand in these age-related process has yet to be determined. Narvaez et al [20] demonstrated that young Cyp27b1 KO mice, which lack 1,25D, exhibit a lean phenotype similar to that of VDRKO mice, but studies on mammary gland and the aging process have yet to be conducted in mice lacking the VDR ligand. Future investigations to specifically address 1,25D and VDR actions in each microenvironment of the breast will be needed to establish direct mechanisms by which vitamin D_3_ signaling regulates adipose tissue, ductal branching and ultimately breast maintenance. In the case of the mammary gland phenotype described here, both direct and indirect effects of VDR signaling in multiple cell types (adipocyte, fibroblasts, epithelial cells) likely contribute to these changes, and aging studies in conditional VDR and cyp27b1 knockout models will offer the best approach for clarification of the underlying mechanisms.

## Materials and Methods

### Animal Maintenance

Wild type and VDR KO mice [42] on the C57Bl6 background were weaned onto and continuously maintained on a “rescue” diet containing 2% calcium, 1.25% phosphorous, and 20% lactose with 2.2 IU vitamin D_3_/g (TD96348, Teklad, Madison, WI). This diet prevents the mineral disturbances and impaired growth associated with VDR ablation [17]. Female mice (10–12 animals per genotype) were sacrificed at 12, 14 and 16 months of age for analysis of mammary gland development. One inguinal gland from each mouse was whole mounted, while the contra lateral gland was formalin-fixed and paraffin embedded. Thoracic mammary glands were harvested and snap frozen for further analysis of protein and RNA. All procedures were approved by the relevant institutional animal care and use committees at the University of Notre Dame (03-92) or the University of Cincinnati (06-03-03-02).

For whole mount analysis, mammary glands were fixed in Carnoy's fixative and stained overnight in Carmine Alum. Samples were dehydrated, cleared in xylene, mounted, and examined on an Olympus SZX12 stereoscope. Whole mounts were used to calculate total area of the fat pad and the degree of branching morphogenesis (assessed by counting the numbers of primary, secondary and tertiary branch points). Primary branches were considered as ducts that arose in the nipple region and extended to the leading edge of the gland, secondary branch points were those that extended from primary ducts and tertiary branches were lateral branches that arose from secondary ducts. The fat pad and lymph node areas were determined by tracing around the outer edges of each tissue and calculating the area with AxioVision software (Zeiss, Inc).

### Indirect Calorimetry

Indirect calorimetry was performed using an Oxymax system (Columbus Instruments, Columbus, OH) in a room with controlled temperature and 12 hour lighting conditions. Mice were placed in individual chambers with free access to food and water. Six weight-normalized WT and VDR KO male mice were placed individually into metabolic isolator chambers, acclimated for three days, reweighed and recorded over a 96 hour time period. Airflow through the chambers was 0.6 L/min. Oxygen consumption (VO_2_) and carbon dioxide production (VCO_2_) for each mouse were measured once per hour for 3.5 minutes (settle time 105 sec, measure time 105 sec). The respiratory quotient (RQ) is the calculated ratio of CO_2_ produced to O_2_ consumed during a given time period. Oxygen consumption (VO_2_), carbon dioxide production (VCO_2_), and energy expenditure were recorded at 3, 6, 9, and 12 months of age in four separate recording periods. The four day measurements were averaged to produce a 24 hour measurement for each mouse, and the six individual mouse data sets were averaged to provide a representative data set for the 24 hour time period for each genotype.

### Histology and Immunohistochemistry

Formalin fixed mammary glands were embedded in paraffin, sectioned at 5 µM, and stained with hematoxylin and eosin Y for routine histological assessment. To detect VDR, formalin fixed paraffin embedded sections were incubated in 2 N HCl at 37°C for 20 minutes. After rinsing in PBS for 5 minutes, slides were incubated overnight with a rat monoclonal antibody directed against VDR (clone 9A7, Neomarkers) at a dilution of 1∶60, followed by incubation with anti-rat secondary antibody at a dilution of 1∶200. To detect estrogen receptor α (ER) and progesterone receptor (PgR), slides were pretreated using citrate buffer (pH-6.0) heated to boiling in a pressure cooker for 15-20 minutes and incubated for 1 hr with mouse ERα (clone 6F11, Novocastra) or PgR (clone AB-7, NeoMarkers) antibodies and detected with the M.O.M. kit (Vector Laboratories) according to manufacturer's directions. To detect smooth muscle actin, slides were digested using a 0.1% trypsin solution for 10 minutes and incubated with a mouse antibody to smooth muscle actin (clone 1A4, Sigma) for 1 hr at room temperature and detected with the M.O.M. kit as above. For all antibodies, sections were counterstained with Harris modified hematoxylin (Fisher Scientific).

### Serum Hormone and Calcium Assays

Blood was removed by cardiac puncture for analysis of serum calcium, leptin and estradiol. Calcium was determined with a colorimetric assay kit (Sigma) according to manufacturer's directions. Leptin was determined using an ELISA kit from Linco Research (St. Charles, MO) and 17β-estradiol radioimmunoassays were conducted with a reagent kit from DiaSorin (Stillwater, MN).

### Real Time Quantitative PCR

Total RNA was isolated from 90–150 mg of frozen thoracic mammary gland with Trizol reagent (GibcoBRL). Independent mammary gland RNA preps from five mice of each genotype were made at the time points indicated in the figure legends. After concentration and purity of the RNA was determined by spectrophotometry, total RNA was reverse transcribed with Taqman Reverse Transcription Reagents (N808-0234, Applied Biosystems). Three independent 1.5 ug cDNA stocks were generated from each RNA sample, and each was independently analyzed in duplicate (60 ng of cDNA/well) using the Taqman PCR Core Reagent Kit (N808-0228, Applied Biosystems) and specific primer and probe sets. Gene expression levels were normalized against 18S RNA, and reported as relative gene expression. For data presentation, duplicate values from each run were averaged, and triplicate values were then averaged to generate one value for each animal. The final data is expressed as the mean ± standard error of five animals/time point.

### Western blotting

Thoracic mammary glands (100 mg) from three animals of each genotype per time point were homogenized in Laemlli buffer containing phosphatase and protease inhibitors [43], separated by SDS-PAGE, transferred to nitrocellulose, and blocked with 5% milk. Immunoblotting was performed with antibodies against VDR (clone 9A7, Neomarkers), Bax (N-20, Santa Cruz Biotech), Caspase-3 (Cell Signaling), E-cadherin (clone 36, BD Biosciences), Occludin (clone 19, BD Biosciences), AKT (Cell Signaling), and estrogen receptor á (clone 6F11, Novocastra). An antibody to GAPDH (clone 6G5, Biogenesis) was used as a loading control. All blots were visualized by enhanced chemiluminescence using products from Pierce.

### Statistical Evaluation

Data are presented as mean ± standard error, with the number of analyses for each mean indicated. Data were analyzed by Student's t test, and means were considered significantly different if a p value less than 0.05 was obtained. All statistical evaluations were performed with Instat software (GraphPad Software, Inc., San Diego California USA, www.graphpad.com).
